# Hybrid scheme for modeling LFPs from spiking cortical network models

**DOI:** 10.1186/1471-2202-14-S1-P119

**Published:** 2013-07-08

**Authors:** Espen Hagen, Maria L Stavrinou, Henrik Linden, Tom Tetzlaff, Sacha van Albada, David Dahmen, Markus Diesmann, Sonja Gruen, Gaute T Einevoll

**Affiliations:** 1 Dept. of Mathematical Sciences and Technology, Norwegian University of Life Sciences, Aas, 1432, Norway; 2 Dept. of Computational Biology, Royal Institute of Technology (KTH), Stockholm 10044, Sweden; 3 Inst. of Neuroscience and Medicine (INM-6) and Inst. for Advanced Simulation (IAS-6), Jülich Research Center and JARA, 52425 Jülich, Germany

## 

**Figure 1:**
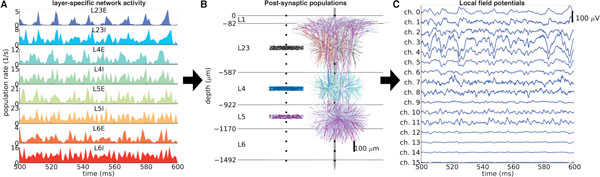
**Schematic illustration of the hybrid scheme**. (**A**) Spiking activity generated in network simulations using single-compartment neurons [2] are used as input to multicompartmental neuron models to generate LFPs (**B**). LFP contributions from each postsynaptic population are calculated and superimposed (**C**).

While recordings of extracellular potentials (EP) remain a common method for experimentally measuring neural activity, the interpretation of the low-frequency part, the *local field potential *(LFP), is not straightforward. Cortical LFPs seem to mainly stem from synaptic inputs, but the net LFP signal from several contributing laminar populations is difficult to assess, as the individual contributions will depend on their locations, the morphologies of the postsynaptic neurons, the spatial distribution of active synapses, and the level of correlations in synaptic inputs [1]. While most comprehensive cortical-network simulations are done with single-compartment models [2], multicompartmental neuronal modeling is in general required to calculate LFPs [1]. Here we present a hybrid LFP modeling approach where a network of single-compartment LIF neurons generates the spiking activity (Figure 1A), while detailed multicompartment neuronal models are used to calculate the accompanying LFP (Figure 1B-C). Our model describes a 1mm^2 ^patch of cat V1, and we incorporate spatially specific pre- to post-synaptic inter- and intra-layer connectivity constrained by experimental observations [3] using reconstructed neuron morphologies of excitatory and inhibitory neurons in layers L2/3-L6 with passive membrane properties. Model specifications of neuron and synapse numbers within populations are taken from [2], while spatial connectivity profiles are based on [3]. Our hybrid simulation framework allows detailed analysis of how the LFP correlates with network activity and connectivity, and how spatially specific synapse distributions influence the LFP. Spiking network simulations [2] were implemented in NEST (http://www.nest-initiative.org), while simulations of LFPs from morphologically realistic neurons used LFPy (compneuro.umb.no/LFPy) along with NEURON [4].
